# Improvement of Hand Hygiene Compliance Using the Plan-Do-Study-Act Method: Quality Improvement Project From a Tertiary Care Institute in Bihar, India

**DOI:** 10.7759/cureus.25590

**Published:** 2022-06-02

**Authors:** Amit Kumar, Rakesh Kumar, Anand K Gupta, Sunil Kishore, Manish Kumar, Rizwan Ahmar, Jayant Prakash, Shambhavi Sharan

**Affiliations:** 1 Pediatrics, Indira Gandhi Institute of Medical Sciences, Patna, IND

**Keywords:** run chart, pdsa cycle, quality improvement, nosocomial infection, hand hygiene

## Abstract

Background

Hospital-acquired infections (HAIs) are the most severe complications of intensive care stay, especially in pediatric patients. Proper hand hygiene (HH) is the cheapest, simplest, but often neglected method to prevent HAIs. The World Health Organization (WHO) has formulated and promoted a standardized recommendation for HH. Both the WHO and the Centers for Disease Control and Prevention (CDC) recommend the use of soap and water for handwashing whenever there is visible dirt on the hands. In all other situations, an alcohol-based hand rub is an effective alternative. The quality improvement (QI) methodology has been widely followed in many countries to improve basic and advanced healthcare systems. The QI strategy follows the plan-do-study-act (PDSA) method.

Methodology

This quasi-experimental (pre- and post-intervention), prospective, QI study was conducted at the neonatal intensive care unit and pediatric intensive care unit of the pediatrics department in a tertiary care hospital in Bihar, India. A QI team was formed. The study was divided into four phases. WHO charts for assessing HH compliance were used for observation and data collection. The EQUATOR Checklist (Squire Checklist) was used to accurately report the QI work. Epi Info™ (version 7.2.5) was used for statistical analysis. The chi-square test was used to measure the statistical difference between pre- and post-intervention HH compliance (proportions).

Results

In the pre-intervention phase, a total of 106 HH opportunities were observed. The HH compliance at this stage was 40.6%. The QI team conducted several meetings, and a root cause analysis was performed with the help of the Fishbone diagram. It was decided to target three probable causes, namely, (a) less awareness, (b) inconvenient locations of hand rub dispensers, and (c) forgetfulness. The QI team decided to run three PDSA cycles. In the last phase, 212 HH opportunities were observed with a compliance percentage of 69.8%. There was a significant improvement when data of pre- and post-intervention HH compliance were compared in all categories of healthcare workers (HCWs), except doctors, where the improvement was not statistically significant. When the cumulative data of all subtypes of HCWs were analyzed, there was a significant improvement (p < 0.0001). Run charts and box plots were used for the easy depiction of the results.

Conclusions

Adopting proper HH methods remains the most effective way of preventing nosocomial infections, especially in intensive care units. We used the WHO model of HH in our study. The pre-intervention HH compliance was 40.6%. QI methodology using root cause analysis and implementation of three PDSA cycles were used to increase the HH compliance percentage. Post-intervention HH compliance increased to 69.8% and the effect was sustained. The study highlights the usefulness of the QI methodology in bringing small but important changes in clinical practice for better patient care.

## Introduction

Hospital-acquired infections (HAIs) are the most serious complications of intensive care stay, especially in pediatric patients. HAI leads to morbidity, mortality, prolonged intensive care unit (ICU) stay, and increased financial burden to the family and nation. Proper hand hygiene (HH) is the cheapest, simplest, but often neglected method to prevent HAIs, i.e., nosocomial infections. Despite being very simple, less time-consuming, and having proper guidelines, the acceptance of effective and complete HH is still not up to the mark [1]. Different studies show compliance to HH ranging between 30% and 75% [2,3].

Ignaz Semmelweis is considered the father of handwashing. The World Health Organization (WHO) has formulated and promoted a standardized recommendation for HH. The WHO has identified the following five important moments to practice HH: moment 1, before touching a patient; moment 2, before a clean/aseptic procedure; moment 3, after body fluid exposure risk; moment 4, after touching a patient; and moment 5, after touching patient surroundings [1].

Both the WHO and the Centers for Disease Control and Prevention (CDC) recommend the use of soap and water for handwashing whenever there is visible dirt on the hands. In all other situations, an alcohol-based hand rub is an effective alternative. The accepted duration for proper handwashing is 40-60 seconds when using soap and water. With alcohol-based formulations, 20-30 seconds are required for proper HH. Concomitant use of soap with water and alcohol-based hand rub is not recommended [1,4]. It is of utmost importance to follow the proper HH method and for the proper duration to decrease the incidence of HAIs. There is a definite need for improved HH compliance among healthcare workers (HCWs) to prevent HAIs and cross-infection among patients as the hands of HCWs infected with microorganisms are the most potent source of HAIs. Simple measures such as teaching, lectures, videos, and demonstration of proper HH methods can be effective for improving compliance. Furthermore, informed observation of the HH procedure has been found to increase HH compliance [5].

Quality improvement (QI) is defined as the combined and unceasing efforts of everyone involved in healthcare, including providers, patients and their families, researchers, planners, and administrators to make changes that will lead to better patient outcomes, better health system performance, and better professional development [6]. Starting in Japan in 1962, the QI methodology has been widely followed in many countries to improve basic and advanced healthcare systems. The QI strategy works according to the plan-do-study-act (PDSA) method. Using the fishbone charts, we first plan what is the problem and what can be done for solving the problem. Pre- and post-intervention outcomes are compared to see if the interventions were beneficial or not. If found beneficial, we try to incorporate the changes in our routine practice for sustained improvement in healthcare. Usually, there is a QI team with a leader, instructors, and observers. The roles can be assigned to any staff, seniority does not count here.

The EQUATOR Checklist (Squire Checklist) is generally used to report the QI work accurately [7]. Many neonatal intensive care units (NICUs) and pediatric intensive care units (PICUs) are regularly using the QI methodology to improve simple but effective interventions such as HH, Kangaroo mother care, early breastfeeding, and prolonging the durability of intensive care instruments [8].

Maintaining proper compliance for a simple but effective procedure like HH can be a difficult task. Therefore, a QI project was started to improve HH compliance at the NICU and PICU of a tertiary care hospital in Bihar, India. A quasi-experimental pre- and post-intervention study to examine the efficacy of the QI methodology was also done. There is no such study from our region, thus justifying the need for the study. Moreover, a QI project may instill acceptance of the methodology for day-to-day improvement in health care, leading to better patient care.

## Materials and methods

Study design and participants

This quasi-experimental (pre- and post-intervention), prospective, QI study was conducted at the NICU and PICU of the Paediatrics Department, Indira Gandhi Institute of Medical Sciences (IGIMS), Patna, Bihar, India. IGIMS, Patna is a tertiary care teaching institute with an eight-bedded NICU and a 16-bedded PICU. The study was conducted between December 2020 and April 2021. Ethical clearance was taken from the institutional ethics committee (1893/IEC/IGIMS/2020). All HCWs including doctors, nursing staff, nursing students, paramedical staff, and helpers (hospital attendants) posted in NICU or PICU and giving consent were included in the study. The identities of all the participants were concealed.

Sample size

According to the Public Health Ontario HH compliance and observation analysis and observation standards, it is estimated that a minimum of 50 HH opportunities must be observed (if the number of beds used for the study is <50) for the study to show any significant result [9,10]. A total of 106 HH opportunities were observed in the pre-intervention phase of the study in the first month of the study, i.e., December 2020, and 452 HH opportunities were studied subsequently over the next 14 weeks as three PDSA cycles were sequentially introduced each month. Data of 212 HH opportunities, which were observed after the introduction of the third intervention, was used as the final post-intervention data to be compared with the pre-intervention data. Weekly analysis of the data was also done to prepare a Run diagram.

Methods

A QI team including two consultants, two nursing staff, and two residents was formed. A one-time written consent was obtained from all HCWs posted in NICU or PICU, or involved in patient care, directly or indirectly. HCWs were categorized into five basic groups, namely, (a) doctors, (b) nursing staff, (c) nursing students, (d) technicians, and (e) hospital attendants and helpers.

The whole team was first trained and retrained as per requirements with the WHO HH module. Doctors were responsible for training, logistics, and monitoring the progress of the study. Nurses and residents were responsible for observing HH opportunities in the prescribed proforma. Predesigned proforma (HH observation form and compliance calculation form) available on the WHO website were used for the study [1].

As suggested by the WHO, observations were done overtly, with participants knowing that they may be observed anytime for HH methods [1]. Identities of HCWs being observed were hidden. To avoid disturbance in routine patient care, the QI team did observations during their off-hours as per their convenience. We tried that all the duty shifts, including night shifts, were equally covered for the observations. Utmost care was taken not to disturb HCWs during observation of HH compliance. One observation session lasted for about 20-30 minutes during which up to three HCWs were observed. If there were more than one HH opportunity, each was marked separately. The QI team tried to observe at least 25 HH opportunities/week.

When HCWs completed the steps of HH at proper moments, properly, for an appropriate duration, only then it was marked as successful completion. Otherwise, it was marked as a negative result.

The study was divided into the following four phases: phase 1, the pre-intervention phase (December 2020); phase 2, after the introduction of the first intervention with the ongoing PDSA cycle (January 2021); phase 3, after the introduction of the second intervention and PDSA cycle (February 2021); and phase 4, after the introduction of the third intervention and PDSA cycle (March-April 2021).

To avoid bias, observers were excluded from the analysis of the study. The main aim of the study was to implement a QI methodology for improving and sustaining HH practice and further use the QI method in other domains of day-to-day practice.

Statistical analysis

WHO charts for assessing HH compliance were used for observation and data collection [1]. The EQUATOR Checklist (Squire Checklist) was used to report the QI work accurately [7]. HH compliance was calculated in both the pre- and post-intervention phase. The data were entered in MS Excel software. MS Excel was used to make graphs, run diagrams, and Box plot charts. Epi Info™ (version 7.2.5) was used for statistical analysis. The chi-square test was used to measure the statistical difference between pre- and post-intervention HH compliance (proportions). P-value was considered significant if values obtained were ≤0.05.

## Results

After obtaining ethical clearance from the institutional ethical committee, the QI project was started in December 2020.

Phase 1 (pre-intervention stage)

The pre-intervention observation of HH compliance was done by the QI team in the last two weeks of December 2020. In this phase, a total of 106 HH opportunities were observed. The five moments of HH opportunities observed were (a) before touching a patient, (b) before clean/aseptic procedures, (c) after body fluid exposure/risk, (d) after touching a patient, and (e) after touching the patient’s surroundings. Each HH opportunity was marked separately. The HH compliance in this stage was 40.6% when data of all categories of HCWs were pooled. The observations of the pre-intervention phase are summarized (Table 1).

**Table 1:** Observations of phase 1 (Pre-intervention stage) CI: confidence interval; HCW: healthcare worker; HH: hand hygiene

Category of HCWs	HH opportunities (No.)	Successful HH completion (No./Percentage/95% CI)
Doctors	26	10 (38.5%) (20.2-59.4)
Nursing staff	29	16 (55.2%) (35.6-73.5)
Nursing students	17	7 (41.2%) (18.4-67.0)
Technicians	16	4 (25%) (7.0-52.0)
Health attendants	18	6 (33.3%) (13.3-59.0)
Total	106	43 (40.6%) (31.1-50.5)

**Figure 1:**
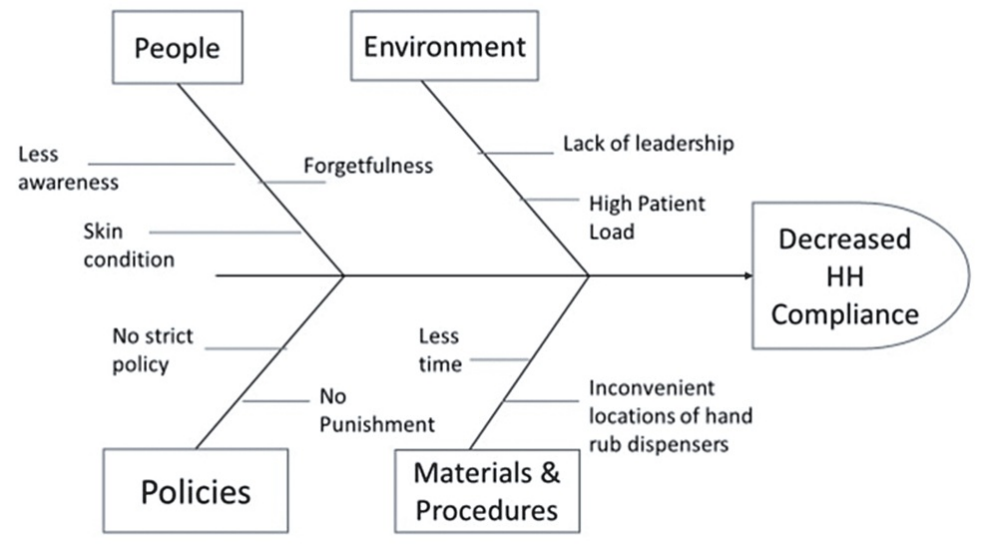
Fishbone diagram showing the root cause analysis. HH: hand hygiene

After the completion of the pre-intervention stage, the QI team conducted several meetings and with the advice of senior teachers and nurses, a root cause analysis was done with the help of the Fishbone diagram (Figure 1).

As this was a simple QI study with no intention to do a major policy change or put a major financial burden, the team decided to act on factors responsible for decreased HH compliance and easily amenable to change. It was decided to target three probable causes, namely, (a) less awareness, (b) inconvenient locations of hand rub dispensers, and (c) forgetfulness. To address these causes, the QI team decided to run three PDSA cycles, each cycle lasting for approximately one month.

Phase 2 (first PDSA cycle): January 2021

The first intervention was teaching sessions for all HCWs posted in or visiting PICU/NICU. The teaching materials and videos were as per the guidelines of WHO and Facility-Based Newborn Care (FBNC). Sessions were divided into small classes which lasted one week. Posters regarding five moments of HH, HH procedure, and the importance of HH were displayed at multiple places.

After the teaching and motivation sessions, overt observation of HH opportunities was again started. This month, 102 HH opportunities were observed by the QI team. There was a definite improvement in HH compliance from 40.6% in the pre-intervention stage to 52.9% at the end of the first PDSA cycle.

Phase 3 (second PDSA cycle): February 2021

The second intervention was to target the lack of proper hand rub dispensers. To tackle this issue, two automated hand rub dispensers were installed each in PICU and NICU at suitable locations. In this cycle, 118 HH opportunities were observed. The HH compliance showed a very little improvement to 54.2%.

Phase 4 (third PDSA cycle): March-April 2021

**Figure 2:**
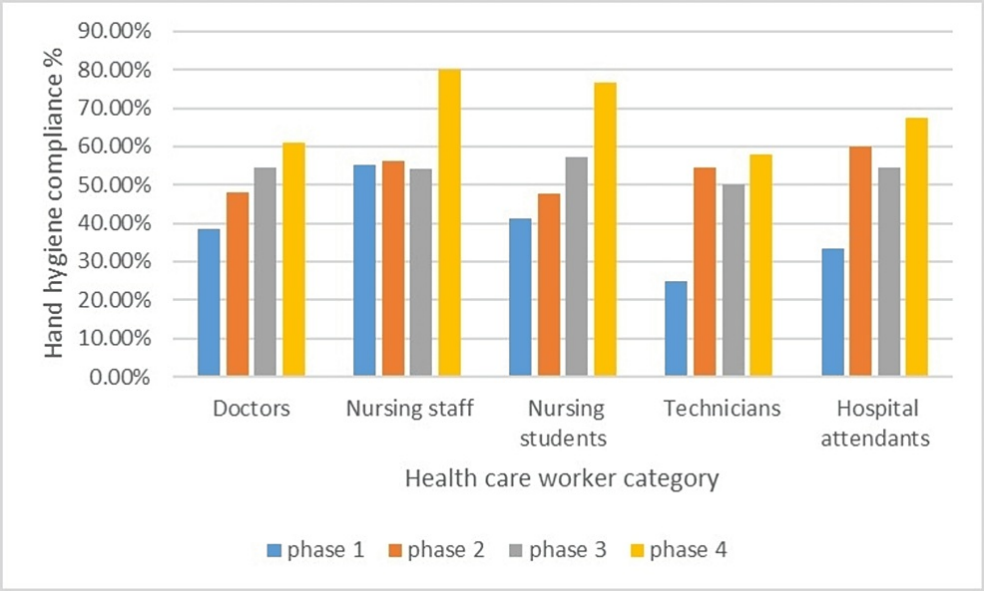
HH compliance in different phases of the study. HH: hand hygiene

Forgetfulness for HH is considered a major cause of poor HH compliance, especially when there is an opportunity to perform HH in between touching two patients. To overcome this problem, one hospital attendant per shift was given the responsibility of playing a pre-recorded, short voice message regarding the importance of HH in the prevention of infection. We used small Bluetooth speakers to play the message. Speakers were connected to desktops placed in NICU and PICU and were kept at appropriate places so that they can be heard sufficiently in handwashing areas, but should not cause disturbance in the working areas. In day shifts, messages were played every one to two hours, and in the night shifts, less frequently at appropriate times. In addition, earlier interventions were also re-emphasized. The last phase of the study was continued for six weeks. Voice prompt, and re-emphasizing earlier measures, showed a marked effect in improving HH compliance this time. A total of 212 HH opportunities were observed with a successful compliance percentage of 69.8% (148 successful completion of HH procedure). HH compliance of all phases as observed in different categories of HCWs is summarized (Figure 2).

**Figure 3:**
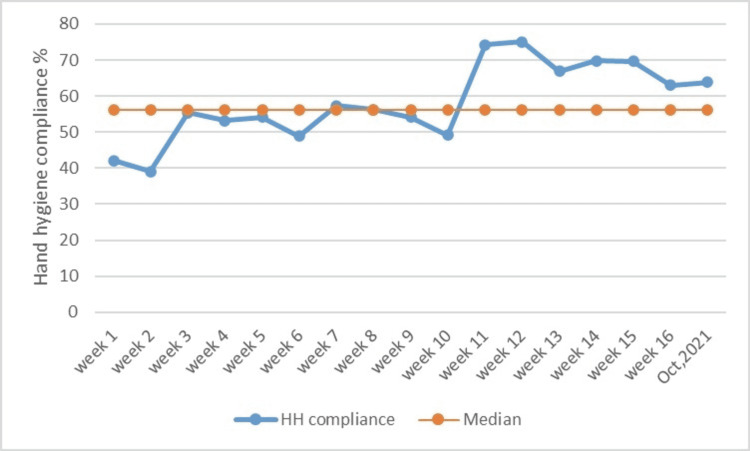
Run chart showing weekly HH compliance data. HH: hand hygiene

After looking at the good results of the QI strategy, the team tried to keep the strategy going on and produce more QI champions. Any new intervention when introduced showed a marked improvement in HH compliance. But the effect reduced over time suggesting the need for continuous motivation and monitoring. One week of HH compliance observation was again done in October 2021 to examine the long-term effect of QI interventions. HH compliance observed then was 63.8%. Though there was a gradual decline in HH compliance over time, it was always better than the acceptable cut-off of 60% and the study median of 56.2% (Figure 3).

A run chart is a very good method for assessing the effect of QI projects. The data in the run chart clearly shows most of the post-intervention values of HH compliance above the median, showing a constructive effect on the healthcare practice. There was a significant improvement when data of pre- and post-intervention HH compliance were compared in all categories of HCWs, except doctors, where the improvement was not statistically significant. The percentage of HH compliance among doctors increased, but it was not significant. The reason for this finding may be fewer doctors observed for HH opportunities. When the cumulative data of all subtypes of HCWs were analyzed, there was a significant improvement (p < 0.0001) (Table 2).

**Table 2:** comparison between pre-and post-intervention HH compliance CI: confidence interval; HCW: healthcare worker; HH: hand hygiene

Category of HCWs	Pre-intervention HH compliance (percentage/95% CI)	Post-intervention HH compliance (Percentage/95% CI)	*X*^2^	P-value
Doctors	38.5% (20.2-59.4)	61.1% (46.8-74.1)	3.561	0.059
Nursing staff	55.2% (35.6-73.5)	80.1% (68.6-89.5)	6.032	0.014
Nursing students	41.2% (18.4-67.0)	76.7% (57.7-90.0)	5.799	0.016
Technicians	25% (7.0-52.0)	57.8% (36.9-76.6)	4.198	0.04
Health attendants	33.3% (13.3-59.0)	67.5% (50.8-81.4)	5.818	0.015
Total	40.6% (31.1-50.5)	69.8% (63.1-75.9)	25.04	<0.0001

**Figure 4:**
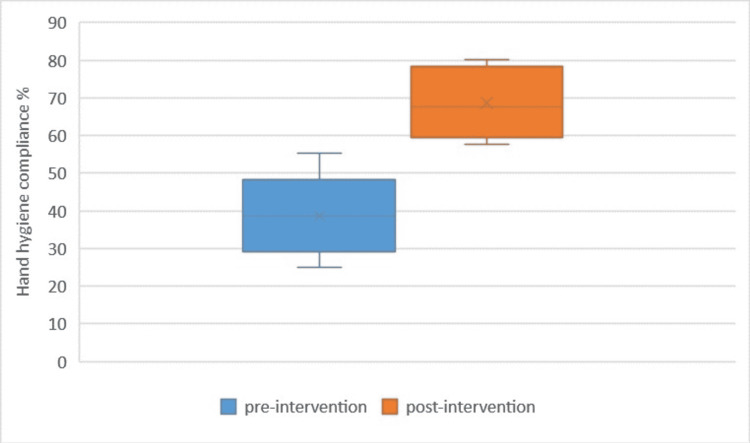
Box plot to show the difference between pre- and post-intervention HH compliance. HH: hand hygiene

Comparative data of pre- and post-intervention HH compliance is shown in a Box plot (Figure 4).

## Discussion

The WHO has provided a well-established, easy-to-follow guideline regarding the moments of HH, methods of HH, and how to measure and improve HH compliance [1]. Globally, most studies have followed the guidelines provided by the WHO to measure HH compliance, as was done in our study. Proper HH remains the most effective method for preventing HAIs, especially in ICUs. Despite its well-established efficacy, compliance with HH remains low. Our study showed a baseline pre-intervention HH compliance rate of 40.6% when composite data of all categories of HCWs were analyzed. This data is comparable with many other studies. Engdaw et al. in their study found HH compliance to be 14.9% [11]. Most studies have shown a baseline HH compliance rate in the range of 30-60%. Lack of proper knowledge among different categories of HCWs regarding the importance and method of HH can be an important cause of low compliance for this basic procedure, as highlighted in a study from Kuwait [12]. Other causes of low HH compliance can be difficult access to handwashing facilities and hand sanitizers which is still prevalent in low-resource settings. Attitude, lack of time and dedication, and high patient load remain other bottlenecks as far as low HH compliance is concerned.

Globally, most studies have shown poor compliance to essential practices such as HH and significant improvement after simple interventions such as teaching and direct observation. Anwar et al. found overall HH compliance increased significantly from 30.9% (95% confidence interval (CI): 27.2-34.6%) before intervention to 69.5% (95% CI: 65.2-72.6%) post-intervention, with the highest HH compliance rate among nurses compared to physicians and workers (p = 0.001) [5]. In our study, HH compliance improved from 40.6% to 69.8% when pre- and post-intervention data were compared. As in other studies, the highest HH compliance was observed among nurses. The cause of this finding may be the permanent posting of nursing staff in ICUs. Posting of other staff including doctors tends to show more rotation as far as the area of duty is concerned.

Demirel et al. used the PDSA method for improving HH compliance at a private hospital. The overall mean compliance increased from 48% to 60% in the post-intervention phase [13]. Chen et al. also used the QI methodology for improving HH compliance. In their study, HH compliance rates improved over time, with significant improvement between pre-intervention (60.1%) and post-intervention (97.2%) periods (p < 0.001). Nurses (88.3%) exhibited higher compliance than dentists (87.3%), and female (88.4%) HCWs were more likely to perform HH than males (85.6%) (both p < 0.001). Overall HH compliance and observance of the five indications exhibited significant linear increases over time (p < 0.005) [14]. We also used the QI methodology to improve HH compliance. A root cause analysis was done using a fishbone diagram. Three PDSA cycles were used to bring the desired changes. We tried to sustain the improvement in HH compliance by producing more QI champions, continued implementation of changes, and occasional assessment of results. The overall acceptance and enthusiasm toward the QI methodology led to sustained results and paved the way for further QI interventions in other areas of healthcare practices such as KMC compliance, shortening the time from arrival to the admission of patients, and prevention of irrational use of antibiotics. QI studies for the abovementioned changes are implemented or being planned.

We used a run chart to demonstrate the efficacy of PDSA cycles over time. Run charts are a very effective, easy-to-understand method of demonstrating the effect of interventions, very commonly used in quasi-experimental QI studies. The X-axis represents time and Y-axis represents the intervention results. We need to calculate the median of the results to interpret the efficacy of interventions. When six or more consecutive points are above the median line in a run chart, we can say there is a shift in healthcare practice [15].

Box plot was also used to diagrammatically show the difference between pre- and post-intervention HH compliance. In a box plot, the input data are split into quartiles. There is a lower quartile (minimum value), median, and upper quartile (maximum value). Comparing box plots gives a very easy interpretation of changes due to interventions [16]. Our box plot showed significant improvement in post-intervention HH compliance.

This was the first QI study at our ICU, with the intention of improvement in simple healthcare procedures and adopting the QI methodology as a routine. Overall, there was a significant improvement, and it was unanimously agreed that the QI methodology can be used to bring other simple but effective changes.

Our study had a few limitations. Overt observations can lead to observation bias, which is a common drawback of QI studies. Further, the number of observations and the duration of the study could have been more.

## Conclusions

Adopting proper HH methods remains the most effective way of preventing nosocomial infections, especially in ICUs. Neonates are most vulnerable to HAIs, thus highlighting the importance of strict HH compliance. Despite well-known efficacy, HH compliance remains low among HCWs. The WHO has proposed guidelines for HH practices, including monitoring its compliance. We used the WHO model of HH in our study. The pre-intervention HH compliance was 40.6%.

The QI methodology using root cause analysis and implementation of three PDSA cycles increased HH compliance percentage. Post-intervention HH compliance increased to 69.8%, and the effect was sustained by making more QI champions and continued implementation of the changes. The enthusiasm for the QI methodology led to its use in the betterment of other easy-to-change but effective healthcare practices. Adopting the QI methodology remains the major aim of this study.
